# Calcium chloride enhances the delivery of exosomes

**DOI:** 10.1371/journal.pone.0220036

**Published:** 2019-07-22

**Authors:** Hyoeun Kim, Ji-Young Kang, Dasom Mun, Nuri Yun, Boyoung Joung

**Affiliations:** 1 Division of Cardiology, Yonsei University College of Medicine, Seoul, Republic of Korea; 2 Brain Korea 21 PLUS Project for Medical Science, Yonsei University, Seoul, Republic of Korea; 3 Institute of Life Science & Biotechnology Yonsei University, Seoul, Republic of Korea; VIT University, INDIA

## Abstract

Exosomes might have an unimproved potential to serve as effective delivery vehicles. However, when exosomes are developed for therapeutic applications, a method to enhance their delivery is important. This study aimed to evaluate wheather calcium chloride (CaCl_2_) or other chloride compounds could enhance exosome delivery to various cells without causing toxicity. Exosomes were purified from human serum by using the ExoQuick exosome precipitation kit. Isolated exosomes were mixed with CaCl_2_ at concentrations ranging from 100 μM to 1 mM, and then washed using Amicon filter for treating the cells. The delivery efficiency of exosomes and the viability of the cells [HEK 293 (human kidney cells) and H9C2 (rat cardiomyocytes)] were evaluated. Cellular uptake of exosomes was observed using a confocal microscope based on PKH26 labeling of exosomes. CaCl_2_ increased the delivery of exosomes in a dose- and treatment time-dependent manner. In HEK 293 cells, a CaCl_2_ concentration of 400 μM and exposure time of 12 h increased the delivery of exosomes by >20 times compared with controls. In H9C2 cells, a CaCl_2_ concentration of 400 μM and exposure time of >24 h increased the delivery of exosomes by >400 times compared with controls. The viability of both cell lines was maintained up to a CaCl_2_ concentration of 1 mM. However, cobalt chloride, cupric chloride, and magnesium chloride did not change the delivery of exosomes in both cell lines. These results suggest that the use of CaCl_2_ treatment might be a useful method for enhancing the delivery of exosomes.

## Introduction

Exosomes are 40–200nm vesicles secreted by many types of cells [1]. They exist in cell culture medium and in numerous body fluids including plasma and serum [2]. The biogenesis of exosomes involves the fusion of multivesicular bodies enclosing pools of endosomal vesicles with the cell plasma membrane and the subsequent release of the vesicles into intercellular space [3]. These vesicles are known to carry a variety of signaling molecules, including nucleic acids, predominantly mRNA and microRNA, functional proteins, and lipids [4]. Owing to their outstanding cell-to-cell communication characteristic, numerous studies have investigated the role of exosomes in physiological and pathophysiological processes, including immune modulation [5,6], tumor metastasis [7], and neurodegenerative diseases [8,9].

Many studies have suggested that exosomes can be ideal candidates for use as carriers for drug delivery [10,11]. Exosomes have been used as delivery vehicles of small nucleic acids such as micro-RNAs and small interfering RNAs or low-molecular-weight medicines [12,13]. Exosome-liposome hybrid nanoparticles can deliver the CRISPR-Cas9 system in mesenchymal stem cells, and thus can be promising tools in *in vivo* gene manipulation [14]. The organ-specific delivery of exosomes was improved by expressing target peptides with Lamp2 on the surface of exosomes [12,15–17]. Pseudotyping exosomes have been suggested as vehicles for the enhanced delivery of protein reporters and protein therapeutics to target cells [18]. However, a simpler method for enhancing the delivery efficacy of exosomes is needed [19].

To date, calcium chloride (CaCl_2_)-associated transfection methods have been widely used to introduce DNA into mammalian cells, with relatively low cost and low toxicity [20]. However, whether CaCl_2_ can increase the delivery efficiency of exosomes to the target cells has not been evaluated. In a recent study, CaCl_2_ and subsequent heat shock-mediated miR-15a-loaded exosomes showed higher delivery of miR-15a to target cells than miR-15a-electroporated exosome [21]. Taken together, we hypothesized that CaCl_2_ or other chloride compounds can affect the delivery efficacy of exosomes into target cells.

Thus, we aimed to investigate whether exosomes mixed with CaCl_2_ can show improved delivery to cells compared with normal exosomes, thus providing an effective method for drug delivery in the future.

## Materials and methods

### Exosome purification and labeling

Human peripheral blood samples from non-atrial fibrillation patients were obtained at Yonsei University Health System (Seoul, Korea). The study protocol conformed to the principles outlined in Declaration of Helsinki, and was approved by the local ethics committee (YUMC 4-2011-0872). Informed consent was obtained from all patients. The name of ethics committee is as follows: Severance Hospital.

Exosomes were purified using the ExoQuick exosome precipitation kit (SBI System Bioscience, Mountain View, CA, USA), according to manufacturer’s instructions. A 250 μL volume of human serum was mixed with 63 μL ExoQuick solution and incubated overnight at 4°C. After centrifugation (1500*g* for 30 min), the supernatant was discarded and tubes were centrifuged again (1500*g* for 5 min). All traces of fluid were aspirated, then pellets were resuspended in 200 μL phosphate-buffered saline (PBS) [22].

After exosome treatment to cells, the uptake of exosomes by cultured HEK 293 cells (human kidney cells) and H9C2 cells (rat cardiomyocytes) was assessed using a confocal microscope (LSM710; Carl Zeiss GmbH, Jena, Germany). For this evaluation, purified exosomes were labeled using PKH26 dye (Sigma, Germany) according to the manufacturer’s instructions, as described previously [23]. Briefly, exosomes were suspended in 1 mL diluent C containing 5 μM PKH26 and incubated for 5 min. The labeling action was stopped by incubating for 1 min with an equal volume of 1% bovine serum albumin (Bovogen, Melbourne, Australia). The exosomes were washed twice with Amicon ultrafilter (10 KDa cut-off, Millipore, MA, USA) with cold PBS. Thereafter, the exosomes were resuspended in 200 μL PBS.

### Mixture of exosomes and CaCl_2_

To deliver exosomes efficiently to cells, a modified method of CaCl_2_ transfection was developed. PKH26-labeled exosomes (50 μg in PBS) were mixed with CaCl_2_ solution (0.2 M stock). The final volume was adjusted to 150 μL using sterile PBS. The mixture was incubated at 37°C in a shaker for 10 min at 25 rpm, and then the tube was immediately placed on ice. The ExoQuick reagent (30 μL) was added, and the mixture was placed on ice for 30 min. The sample was centrifuged for 3 min at 13,000–14,000 rpm in a microfuge. The supernatant was removed, and the mixture pellet was resuspended in 1 mL PBS. The pellet was washed using Amicon Ultra tubes with cold PBS. Thereafter, the exosomes were resuspended in 200 μL PBS.

### Western blot analysis

Western blot analysis was performed as we described previously [16]. Briefly, ultracentrifuged exosomal pellets were lysed with radioimmunoprecipitation buffer (ATTO, NY, USA) containing a protease inhibitor cocktail (ATTO). The total amount of protein was determined using a 660-nm protein assay (Pierce, MA, USA), and equal amounts (20 μg) of exosomal proteins were resolved using sodium dodecyl sulfate-polyacrylamide gel electrophoresis and transferred to polyvinylidene difluoride membranes. The blots were probed overnight at 4°C with anti-Lamp2 (SC-18822; Santa Cruz Biotechnology, Santa Cruz, CA, USA), anti-CD81 (SC-166029, Santa Cruz Biotechnology), or anti-Alix (SC-99010, Santa Cruz Biotechnology), as indicated. The membranes were then exposed to horseradish peroxidase-conjugated mouse or rabbit anti-mouse secondary antibodies (Santa Cruz Biotechnology), and the results were visualized using chemiluminescence (Advansta, Menlo Park, CA, USA).

### Transmission electron microscopy

A Formvar-carbon-coated electron microscope grid was placed with the formvar side down on top of an exosome drop for approximately 1 min. The grid was removed, blotted with filter paper, and placed onto a drop of 2% uranyl acetate for 15 s. The excess uranyl acetate was removed, and the electron microscope grid was examined and photographed for transmission electron microscopy (TEM). All thin sections were observed under a transmission electron microscope (JEM-1011; JEOL, Tokyo, Japan) at an acceleration voltage of 80 kV. Images were captured with a side-mounted Camera-Megaview III (Soft Imaging System, Münster, Germany) [24].

### Nanoparticle tracking analysis

The number of nanoparticles in serum-derived exosomes was assessed using the Nanosight LM10-HS nanoparticle characterization system (Nanosight Ltd., Amesbury, UK). Three recordings were performed for each sample. The Nanosight Tracking Analysis 3.2 software was then used to analyze the video, and to determine the particle concentration and the size distribution of the particles. Three videos of 10-s duration were recorded for each sample.

### Cell culture

HEK 293 cells and H9C2 cells were cultivated as we described previously [16]. Briefly, HEK 293 cells (Korean Cell Line Bank, Seoul, Korea) and H9C2 cells (American Type Culture Collection, Manassas, VA, USA) were maintained in Dulbecco’s modified Eagle’s medium (Welgene, Daegu, Korea) containing 10% fetal bovine serum (Young In Frontier, Seoul, Korea) and 1% penicillin-streptomycin (Gibco, NY, USA). Cells were cultured in a humidified incubator at 37°C with 5% CO_2_.

### Immunocytochemistry and confocal microscopy

Immunocytochemistry and confocal microscopy were executed as we described previously [16]. After treating HEK 293 and H9C2 cells with or without exosomes for 24 h, the cells were fixed with 4% paraformaldehyde for 60 min at room temperature and washed with PBS. Cell nuclei were stained with 4′6‐diamidino‐2‐phenylindole (Santa Cruz Biotechnology). Fluorescence images were obtained using a Zeiss LSM710 confocal microscope with 2 excitation filters (405 and 543 nm). The data were recorded as serial optical sections, each consisting of 1024 × 1024 pixels, overlaid to distinguish between the separate emission channels, and saved as TIFF (tagged image file format) files. Quantification was performed using Image J program. PKH26 expression and DAPI expression were measured respectively using the RGB function of histogram. The same slide was averaged by taking three different parts, and the number of samples was three.

### Cell proliferation assay

The cytotoxic potential of exosomes was assessed using an MTS [3-(4,5-dimethylthiazol-2-yl)-5-(3-carboxymethoxyphenyl)-2-(4-sulfophenyl)-2H-tetrazolium] assay (Promega, Madison, WI, USA). HEK 293 cells were treated with exosomes in triplicate in a 96-well plate. Cell survival was determined using an enzyme-linked immunosorbent assay plate reader (Molecular Devices, Menlo Park, CA, USA).

### Mouse and NIRF imaging

Male C57BL/6 mice (25 g) were purchased from Orient Bio (Seoul, Korea). All animal experiments were approved by the Institutional Animal Care and Use Committee of Yonsei University College of Medicine (Seoul, Korea), and were conducted in accordance with the National Institutes of Health Guidelines for the Care and Use of Laboratory Animals. We injected 150 μg of PKH26 labeled exosomes per animal. After 24 h, the heart, liver, and spleen of each mouse were harvested and IVIS Spectrum imaging system (PerkinElmer, Waltham, MA, USA) was employed to capture Near-infrared fluorescence (NIRF) images. PKH26-related fluorescence signals were discriminated from the auto-fluorescence signals using Living Image software (PerkinElmer).

### Statistical analysis

Data analyses were performed with Student's t-test between two groups. A p-value of <0.05 was considered statistically significant. All statistical analyses were conducted with SPSS version 23.0 statistical package (SPSS Inc., Chicago, IL, USA).

## Results

### Isolation and characterization of serum exosomes

**Fig 1:**
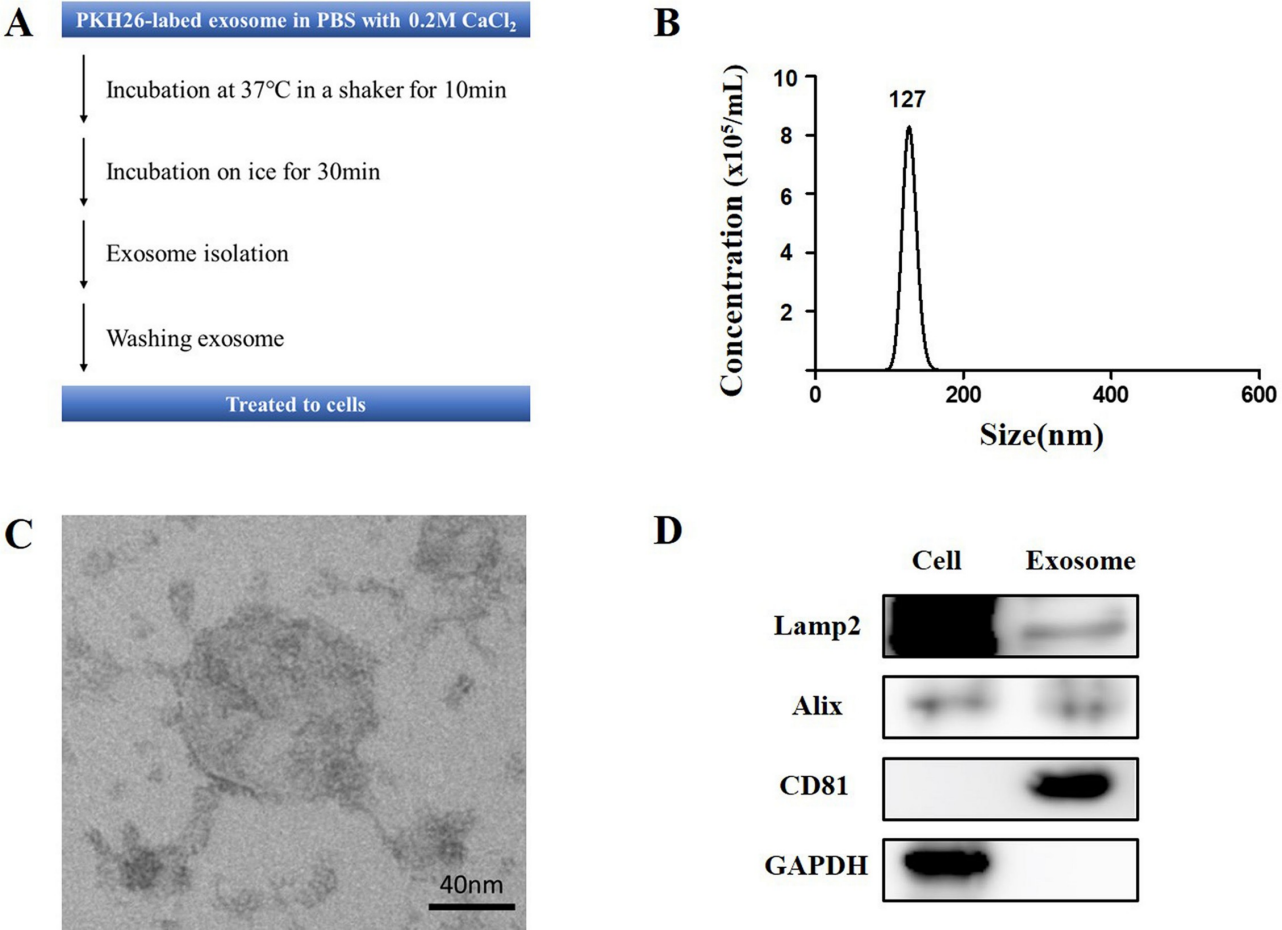
Characterization of exosomes. (A) Study protocol. (B) Nanoparticle tracking analysis of exosomes showing the number and size distribution of particles. (C) Representative electron microscopic image of the exosomes (scale bar, 40 nm). (D) Western blot analysis of isolated exosomes and cell lysate.

The protocol of preparing the mixture of exosomes and CaCl_2_ is shown in Fig 1A. CaCl_2_-associated transfection methods are widely used for introducing DNA into mammalian cells [20]. We used a modified protocol in which incubation at 37°C in a shaker was performed to increase exosome delivery to cells. Exosomes were isolated from peripheral blood by using the ExoQuick reagent, as described in Materials and Methods. The profiles of serum-derived exosomes were characterized using nanoparticle tracking analysis, TEM, and western blotting. According to the Nanosight instrument, the mean particle diameter was 136.6 nm, and mean concentration was 8.59×10^7^ particles/ml (Fig 1B). And structurally intact exosomes were detected by TEM analysis (Fig 1C). There was no significant difference in the size and concentration of exosomes before and after addition of CaCl_2_ when TEM and NTA were compared (S1 Fig). When cell lysate and exosome were examined by western blot analysis, the exosomes were positive for exosomal markers including Alix, CD81, and Lamp2 (Fig 1D). Taken together, the exosomes were successfully purified.

### CaCl_2_ dose-dependent delivery of exosomes to HEK 293 and H9C2 cells

**Fig 2:**
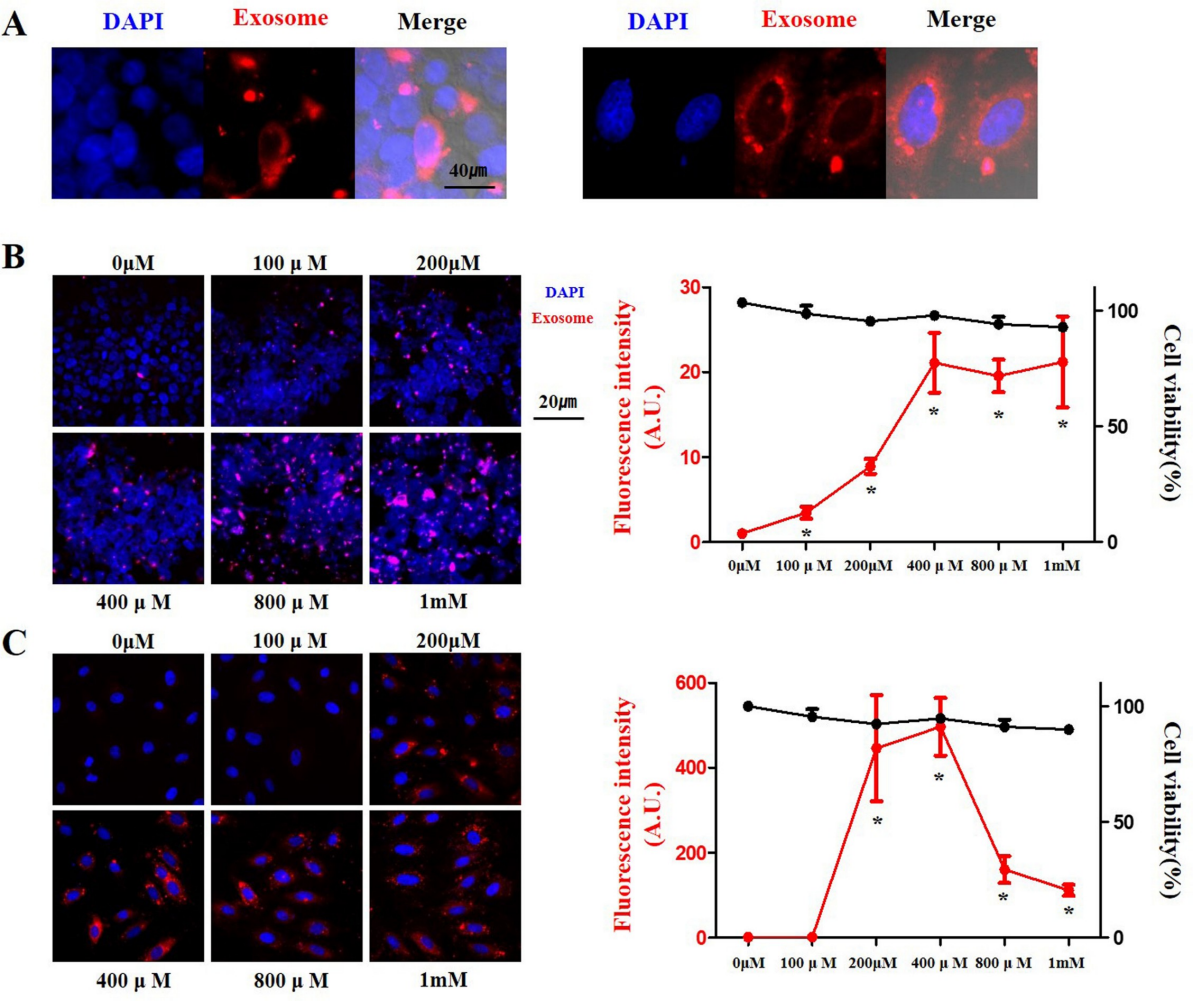
Delivery efficiency of exosomes with CaCl_2_ in a dose-dependent manner. (A) Typical example of delivery of exosomes to HEK 293 cells (left panels) and H9C2 cells (right panels). (B) and (C) Delivery efficiency and cell viability of exosomes labeled with PKH26 (red) at different concentrations of CaCl_2_ in HEK 293 (B) and H9C2 (C) cells. Fluorescent microscopic images (left panels), and fluorescence intensity and cell viability (right panels). The data are presented as the mean ± s.e.m. *P < 0.01.

To identify whether CaCl_2_ treatment improves delivery of exosomes, we added several concentrations of CaCl_2_ to exosomes and these mixtures were incubated at 37°C in a shaker. The mixture of exosomes and CaCl_2_ was used to treat the HEK 293 and H9C2 cells, which were then incubated for 24 h. The most commonly used method for monitoring exosome uptake is to stain exosomal membranes with fluorescent lipid membrane dyes such as PKH26 [25], PKH67 [26], and DiI [27]. In this study, PKH26 was used to label the exosomes and to monitor the uptake of labeled exosomes by cells. The uptake of labeled exosomes by cells was confirmed using a confocal microscope (Fig 2A).

In HEK 293 cells, the delivery of exosomes was dose-dependently increased. At a CaCl_2_ concentration of 400 μM (P < 0.001), the delivery of exosomes increased the most, and then reached a plateau. In H9C2 cells, the delivery of exosomes was significantly increased over 400 times (P = 0.003) at a CaCl_2_ concentration of 200 μM, After peaking at 400 μM, it decreased at higher concentrations (P < 0.001).

In order for exosomes to be used as therapeutic application, there should be no damage to the cells. Thus, we checked cell viability after exosomes incubation. Cell viability was maintained up to a CaCl_2_ concentration of 1 mM in both cells (Fig 2B and 2C). As a result, we found that the CaCl_2_ treatment improved the delivery of exosomes to cells without toxicity.

### Optimal time for exosome uptake by cells

**Fig 3:**
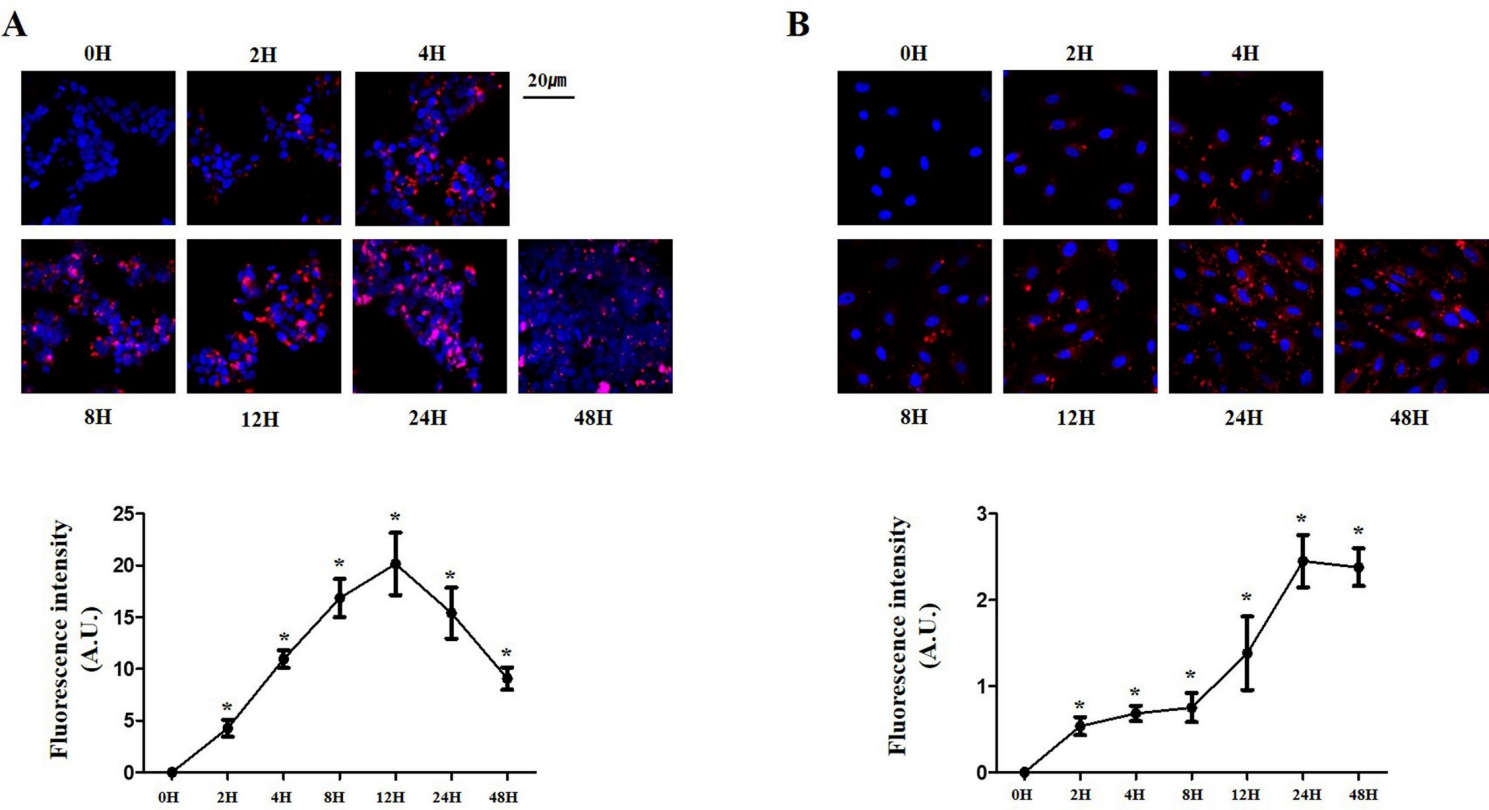
Delivery efficiency of exosomes with CaCl_2_ in a time-dependent manner. (A) and (B) Delivery efficiency of exosomes labeled with PKH26 (red) at different time exposures to CaCl_2_-treated exosomes in HEK 293 (A) and H9C2 (B) cells. Fluorescent microscopic images (upper panels) and fluorescence intensity (lower panels). *P < 0.01.

Because 400 μM was the CaCl_2_ concentration with the maximum delivery of exosomes, we evaluated the cellular uptake of exosomes at different durations of exposure to these CaCl_2_-treated exosomes. In HEK 293 cells, the cellular uptake of exosomes increased as the incubation time increased, with saturation at around 12 h and a decrease after 24 h (Fig 3A, P < 0.0001). In H9C2 cells, the uptake increased as the incubation time increased, with saturation at around 24 h and a decrease after 48 h (Fig 3B, P < 0.0001). Taken together, we found the most efficient concentration of CaCl_2_ and the appropriate treatment time.

### CaCl_2_ is more effective than other chloride compounds

**Fig 4:**
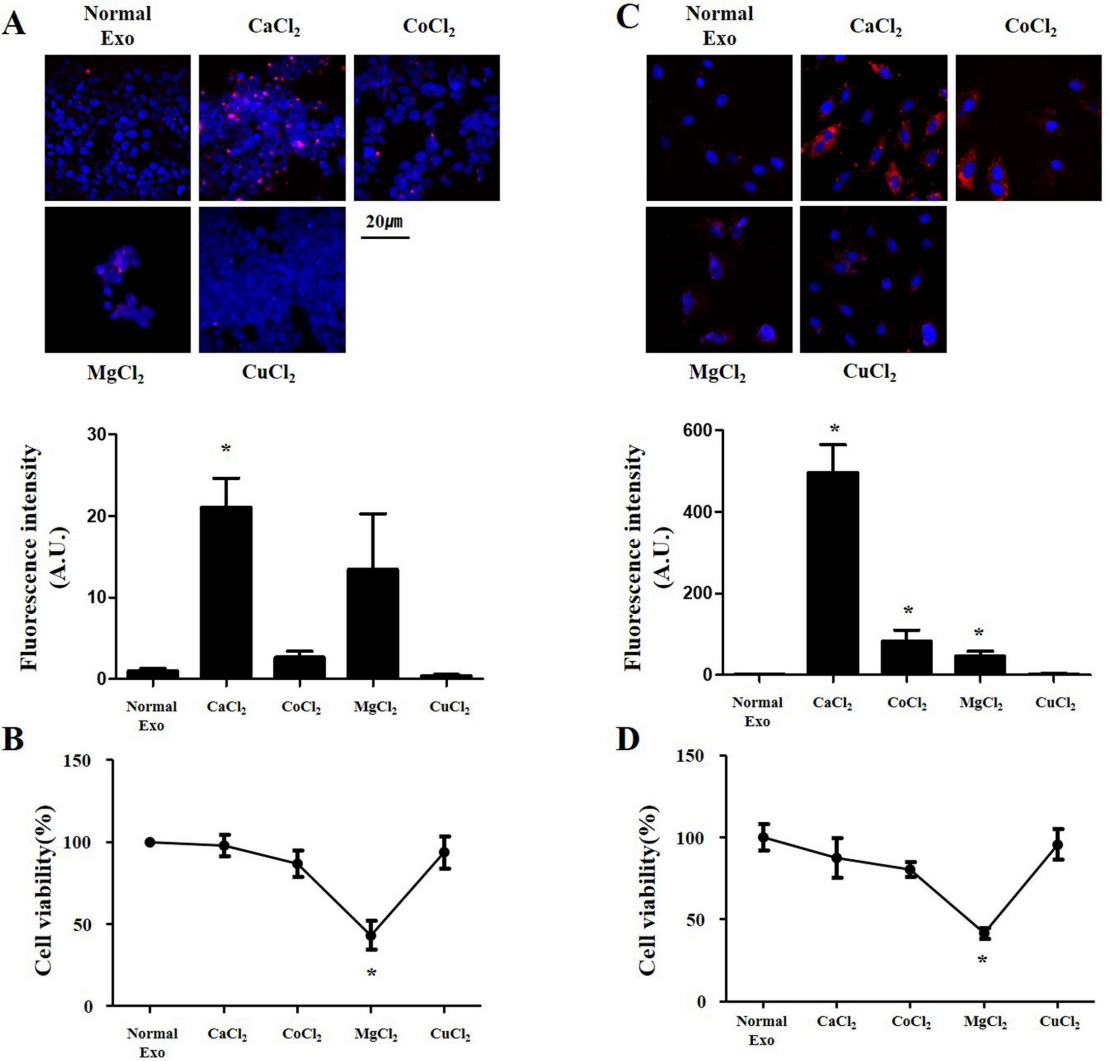
Delivery efficiency of exosomes with chloride compounds. (A, C) Delivery efficiency and (B, D) cell viability of exosomes labeled with PKH26 (red) added with different chloride compounds in HEK 293 (A,B) and H9C2 (C,D) cells. Fluorescent microscopic images (upper panels), and fluorescence intensity and cell viability (lower panels). *P < 0.01.

Next, we assessed whether the cellular uptake of exosomes will be increased by other chloride compounds. Cobalt chloride (CoCl_2_), magnesium chloride (MgCl_2_) and cupric chloride (CuCl_2_) were treated exosome at a concentration of 400μM for 24 hours. Exosome delivery and cell viability were measured by the above method. In HEK 293 cells, the cellular delivery of exosomes was not significantly improved by CoCl_2_ or MgCl_2_, but was rather decreased by CuCl2 (Fig 4A). In H9C2 cells, the cellular delivery of exosomes was improved by CoCl_2_ (P = 0.017) and MgCl_2_ (P = 0.004), but not by CuCl_2_ (Fig 4B). Moreover, the cell viability was significantly decreased by MgCl_2_ in both cell lines. These results show that CaCl_2_ significantly enhances intracellular delivery of exosomes compared to other chloride compounds.

### *In vivo* delivery of CaCl_2_-treated exosomes

We also performed *in vivo* experiments to support the claim that CaCl_2_ enhances the delivery of exosomes. Exosomes were injected via tail vein in mice and sacrificed 24 hours later. The level of PKH26 was measured using In Vivo Imaging System (IVIS) of organs. We confirmed that CaCl_2_-treated exosomes significantly increased the delivery in heart, lung, kidney, and spleen (S3 Fig). Based on these results, we suggest that CaCl_2_-treated exosomes allow higher delivery than normal exosomes *in vivo*.

## Discussion

In this study, we attempted to enhance the delivery efficiency of exosomes by using CaCl_2_, which is commonly used for transfection of DNA into mammalian cells. Exosomes incubated with CaCl_2_ increased delivery efficiency in a dose-dependent manner in HEK 293 and H9C2 cells. The optimum duration of treatment with exosomes added with CaCl_2_ was 12 h for HEK 293 cells and 24 h for H9C2 cells. Finally, it was confirmed that CaCl_2_ significantly increased the intracellular uptake of exosomes when compared with other chloride compounds at the same concentration. On the basis of these results, we suggest that adding CaCl_2_ to exosomes for treating the cells is an efficient method of increasing the delivery of exosomes.

Exosomes are vesicles of endocytic origin released by many cells [28]. They are crucial in distant cell-cell communication because they can enter the circulatory system when they are secreted and can pass through additional biological barriers [29]. R. Liu et al. recent accumulated evidence suggests that these nano-sized vesicles can deliver various RNAs into cells from the natural pathway, to deliver genetic material in organisms [30]. As exosomes are promising for use as vectors in clinical applications owing to their strong biocompatibility, enhancement of exosome delivery is an important research topic [31]. One of the limitations of exosomes is the requirement for high capacity production for clinical use [19,32]. Our findings provide a solution to this limitation.

In the standard method for transforming of *Escherichia coli* with external DNA, cells are known to be suitable for DNA uptake by incubating in ice-cold 100 mM CaCl_2_ [33]. CaCl_2_ assists the interactions between DNA molecules and the cell surface and helps endocytosis of the DNA molecules [34]. We modified this protocol to increase the uptake of exosomes into cells. Also, calcium influx induces endocytosis and exocytosis, and is known to trigger vesicle fusion [35–37]. Therefore, we anticipate that the addition of CaCl_2_ increases the efficiency of exosome delivery. However, more research is needed to confirm these mechanisms. In our study, up to 1 mM CaCl_2_ was used, but no cytotoxicity was observed. Moreover, CaCl_2_ is a common compound used in various research institutes. In this experiment, a low CaCl_2_ concentration was used, thus providing the advantages of low cost and high accessibility. We also compared CaCl_2_ with other chloride compounds. Our results showed that CaCl_2_ was the only common enhancer of exosome delivery between HEK 293 and H9C2 cells. Our findings provide useful technological insights for the development of exosome-mediated drug delivery.

## Supporting information

S1 Fig Characterization of CaCl_2_-Exo. (A) Representative electron microscopic image of CaCl_2_-Exo (scale bar, 40 nm). (B) Size distribution of CaCl_2_-Exo measured from TEM images. (C) Nanoparticle tracking analysis of CaCl_2_-Exo showing the concentration of particles.
(TIF)

S2 Fig Un-cropped Western blots from main Fig 1. (TIF)

S3 Fig *In vivo* delivery of exosomes and CaCl_2_-Exo. (A) Representative NIRF images (overlaid with photograph) of mice organs which received administration of PBS, PKH26-labeled exosomes, or CaCl_2_-Exo. (B) Quantitation of fluorescence intensity in the lesion region. *P<0.05.
(TIF)
