# Supplementary figures and images for: Calcium chloride enhances the delivery of exosomes
Source: PLoS One. 2019 Jul 22;14(7):e0220036. doi: 10.1371/journal.pone.0220036 (PMC6645520; doi:10.1371/journal.pone.0220036)

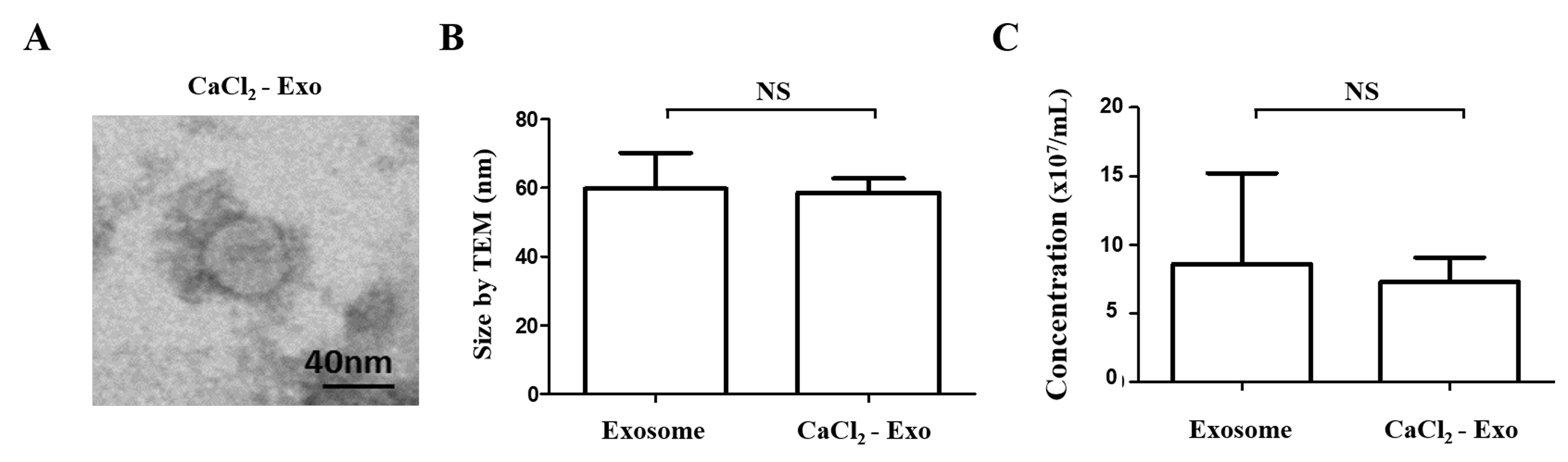

Supplement: S1 Fig — (A) Representative electron microscopic image of CaCl2-Exo (scale bar, 40 nm). (B) Size distribution of CaCl2-Exo measured from TEM images. (C) Nanoparticle tracking analysis of CaCl2-Exo showing the concentration of particles. (TIF) [file pone.0220036.s001.tif]

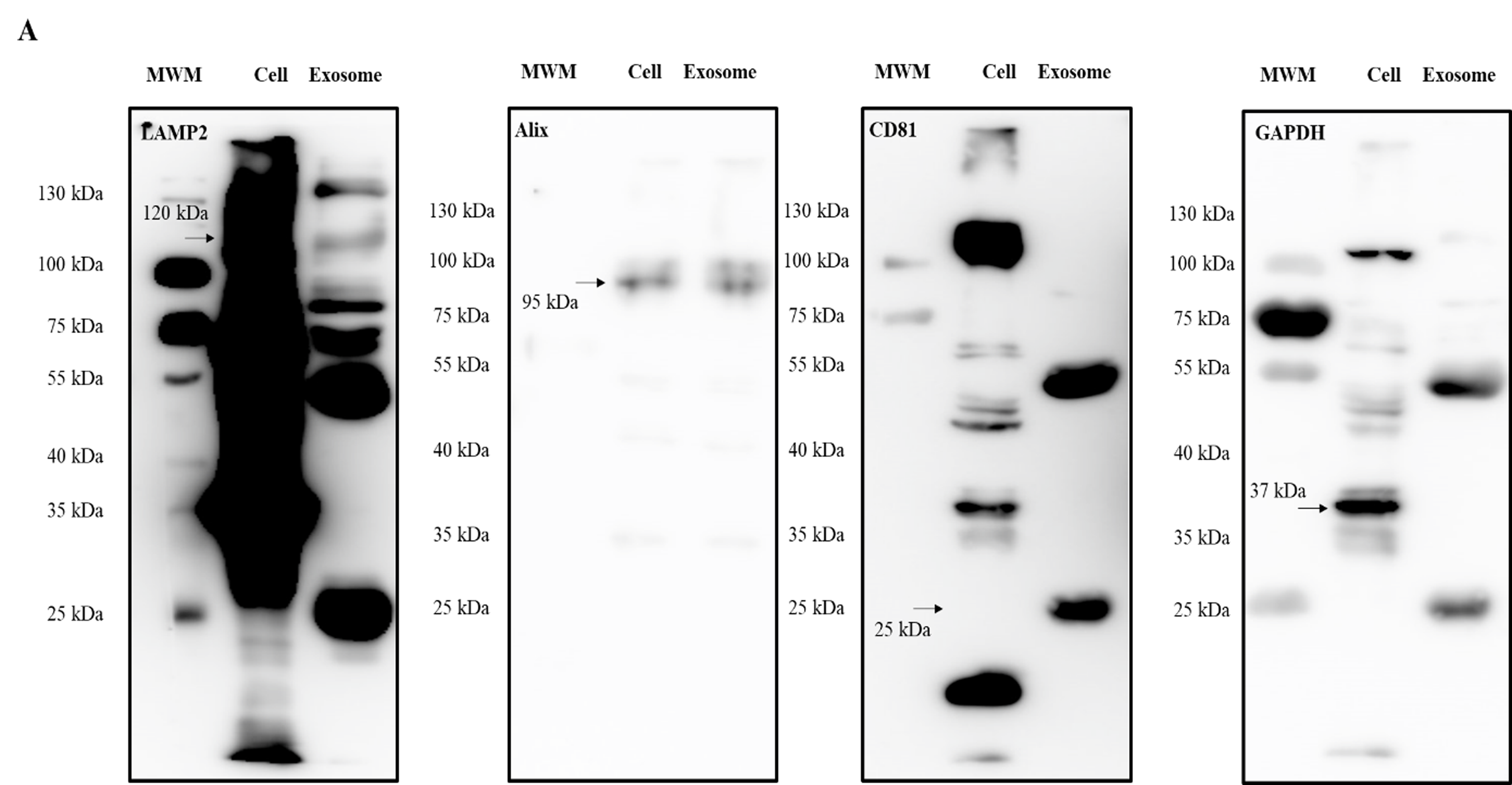

Supplement: S2 Fig — (TIF) [file pone.0220036.s002.tif]

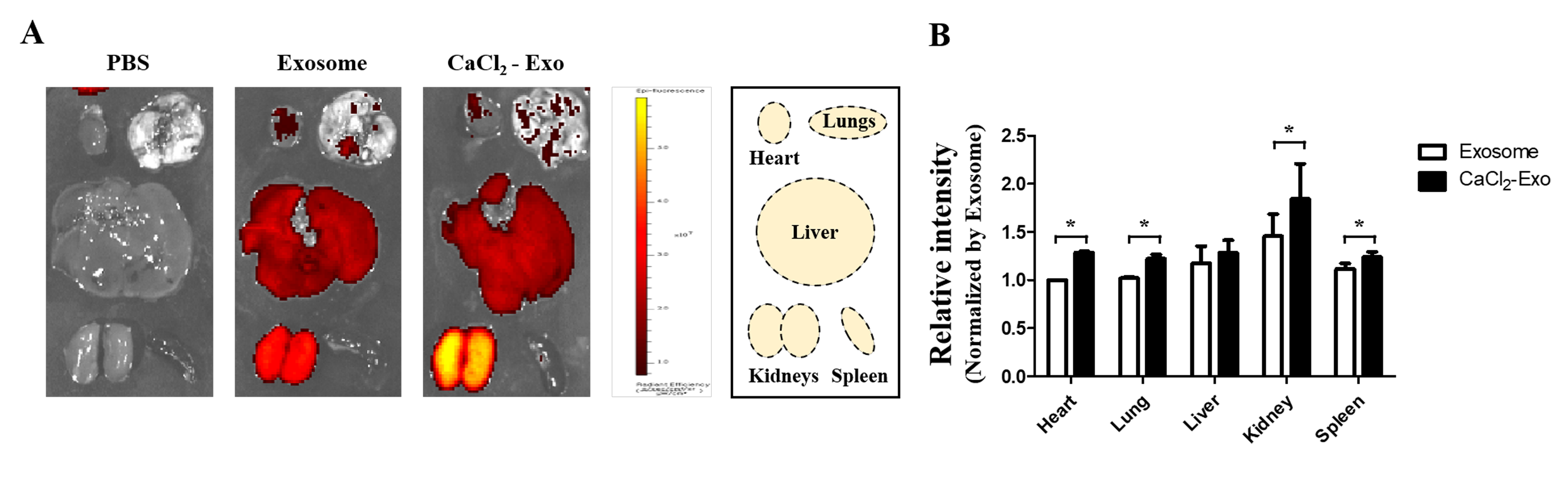

Supplement: S3 Fig — (A) Representative NIRF images (overlaid with photograph) of mice organs which received administration of PBS, PKH26-labeled exosomes, or CaCl2-Exo. (B) Quantitation of fluorescence intensity in the lesion region. *P<0.05. (TIF) [file pone.0220036.s003.tif]
